# Choice of Drug for Malaria Prevention During Pregnancy Does Not Affect Infant Serologic Responses to *Plasmodium falciparum* Erythrocyte Membrane Proteins 1

**DOI:** 10.1093/ofid/ofaf037

**Published:** 2025-01-23

**Authors:** Amed Ouattara, Liana R Andronescu, Matthew Adams, Ankur Sharma, Rie Nakajima, Aarti Jain, Omid Taghavian, Algis Jasinskas, Philip L Felgner, Don P Mathanga, Jobiba Chinkhumba, Miriam K Laufer, Mark A Travassos

**Affiliations:** Malaria Research Program, Center for Vaccine Development and Global Heath, School of Medicine, University of Maryland, Baltimore, Maryland, USA; Malaria Research Program, Center for Vaccine Development and Global Heath, School of Medicine, University of Maryland, Baltimore, Maryland, USA; Malaria Research Program, Center for Vaccine Development and Global Heath, School of Medicine, University of Maryland, Baltimore, Maryland, USA; Malaria Research Program, Center for Vaccine Development and Global Heath, School of Medicine, University of Maryland, Baltimore, Maryland, USA; Vaccine Research and Development Center, Department of Physiology and Biophysics, School of Medicine, University of California, Irvine, Irvine, California, USA; Vaccine Research and Development Center, Department of Physiology and Biophysics, School of Medicine, University of California, Irvine, Irvine, California, USA; Vaccine Research and Development Center, Department of Physiology and Biophysics, School of Medicine, University of California, Irvine, Irvine, California, USA; Vaccine Research and Development Center, Department of Physiology and Biophysics, School of Medicine, University of California, Irvine, Irvine, California, USA; Vaccine Research and Development Center, Department of Physiology and Biophysics, School of Medicine, University of California, Irvine, Irvine, California, USA; Malaria Alert Center, Kamuzu University of Health Sciences, Blantyre, Malawi; Malaria Alert Center, Kamuzu University of Health Sciences, Blantyre, Malawi; Malaria Research Program, Center for Vaccine Development and Global Heath, School of Medicine, University of Maryland, Baltimore, Maryland, USA; Malaria Research Program, Center for Vaccine Development and Global Heath, School of Medicine, University of Maryland, Baltimore, Maryland, USA

**Keywords:** chemoprophylaxis, dihydroartemisinin-piperaquine, infants, pfEMP1, sulfadoxine-pyrimethamine

## Abstract

While sulfadoxine-pyrimethamine has been the primary drug in intermittent preventive treatment in pregnancy, dihydroartemisinin-piperaquine (DP) is being considered as an alternative. DP may lead to lower antimalarial antibodies in the mother, resulting in higher risk of malaria in infancy. We probed cord blood sera collected from women enrolled in a clinical trial of sulfadoxine-pyrimethamine vs DP on a protein microarray containing diverse *Plasmodium falciparum* erythrocyte membrane proteins 1 to measure the impact of intermittent preventive treatment in pregnancy on proteins associated with malaria disease susceptibility. These results suggest that effective maternal malaria prevention may not alter the susceptibility of infants to malaria.

Sulfadoxine-pyrimethamine (SP) has been the drug of choice for intermittent preventive treatment in pregnancy (IPTp) for decades [1], though the development of malaria parasite resistance to SP may limit its use in the near future. The drug combination dihydroartemisinin-piperaquine (DP) is highly effective in treating and preventing malaria, and IPTp with DP reduces malaria burden during pregnancy in clinical trials [2]. Given a sustained posttreatment prophylactic effect, DP is an attractive IPTp combination therapy [3]. Although findings on transferred maternal antibody and malaria risk are mixed [4], recent data suggest that high levels of maternal antibodies to blood-stage antigens have been associated with a reduced risk of malaria in early childhood [5]; by preventing blood-stage disease during pregnancy, DP may paradoxically increase infant risk of malaria by lowering the circulating maternal antibodies to blood-stage malaria infection. Hence, a lower concentration of those antibodies will be transferred across the placenta to protect the infant from malaria infection and disease early in life.

*Plasmodium falciparum* erythrocyte membrane proteins 1 (PfEMP1s) are a family of genes that are frequently linked to susceptibility to clinical malaria disease because they mediate binding of infected erythrocytes to endothelial cells. Expression of PfEMP1 subsets are associated with specific clinical syndromes, including endothelial protein C receptor–binding PfEMP1s in severe disease [6] and CD36-binding PfEMP1s in uncomplicated or asymptomatic malaria [7, 8].

As DP prevents blood-stage malaria infection during pregnancy better than SP, we hypothesize that babies born to mothers who receive DP will have lower serologic responses to blood-stage antigens. To address this hypothesis, we probed cord blood sera from infants born to mothers in a clinical trial comparing IPTp with SP vs IPTp with DP on a customized protein microarray of diverse PfEMP1 features [9] to determine if humoral responses to these key surface proteins differ between the treatments arms.

## METHODS

### Sample Origin and Participants

Samples used in this study originated from a randomized clinical trial (NCT03009526) conducted in southern Malawi. Pregnant women with <28-week gestation who were HIV negative were offered enrollment in the clinical trial and randomized to receive either SP or DP monthly for IPTp until delivery [10]. For mothers who consented to enrollment in the infant study (n = 602), blood was collected from the umbilical cord at delivery (n = 196), with sera stored at −80 °C after centrifugation [10]. The sera were later shipped to the University of Maryland for microarray probing.

### PfEMP1 Microarrays

The microarray was designed by amplifying the open reading frames of 260 *P falciparum* protein features, followed by the cloning and printing of protein features on a chip, as described previously [11]. The protein microarray primarily consisted of a representative set of 166 PfEMP1 features, including 80% of the PfEMP1 domains from the 3D7 reference strain [9]; PfEMP1s from the IT4, HB3, and DD2 reference strains; and PfEMP1s from clinical infections. Microarrays were probed with cord blood sera to measure total IgG responses, and the arrays were scanned with a microarray scanner (ScanArray Express HT; PerkinElmer), followed by fluorescence quantification (ScanArray Express Suite; PerkinElmer).

### Data Analyses

Fluorescence intensity was defined as the raw signal intensity corrected by global median scaling for no-DNA negative control features. The resulting signal for each protein feature was called the median fluorescent intensity (MFI), which was used to assess seroreactivity, defined as the magnitude of microarray fluorescence intensity and serorecognition. Positive serorecognition for an individual sample was defined as an MFI that was 2 SD above the MFI for 10 North American controls that were malaria naive. Group serorecognition for a protein feature was defined as whether a group's MFI distribution was greater than that of the North American control group (Wilcoxon rank sum test). To determine the association between prophylactic regimens given to pregnant women and the serorecognition of PfEMP1s in newborns, a Fisher exact test was performed comparing the 2 IPTp regimens. Seroreactivity differences for each protein feature were compared between treatment arms with a Wilcoxon rank sum test. Presented *P* values are 2-sided without correction for multiple comparisons, with an alpha of .05 per previously described approaches for microarray analyses [9, 12]. All analyses were performed with SAS version 9.4 (SAS Institute), R version 4.2.3, and RStudio.

## RESULTS

**Figure 1.**
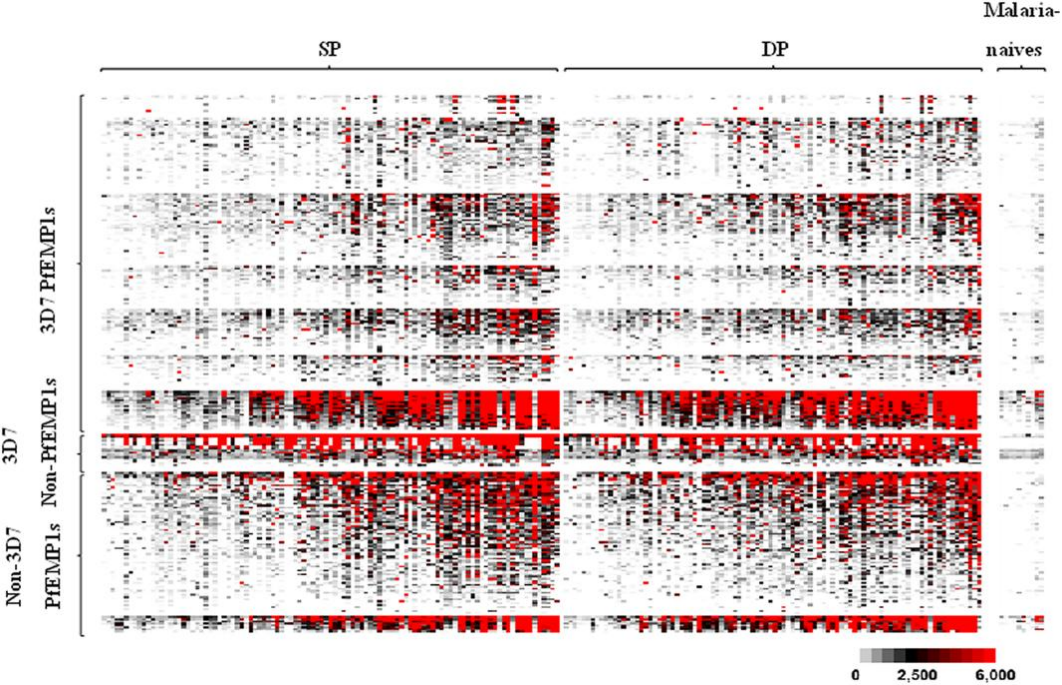
Heat map of seroreactivity against 247 malaria protein features in children born to pregnant women undergoing intermittent preventive treatment in pregnancy with SP or DP. Columns: Individuals are arranged according to treatment arms (SP and DP) and negative controls (malaria-naive North American adults). Rows: Antigen features are arranged with increasing signal intensities. The color indicates the magnitude of the response to each antigen, ranging from gray (weak) to black (intermediate) and red (intense). DP, dihydroartemisinin-piperaquine; PfEMP1s, *Plasmodium falciparum* erythrocyte membrane proteins 1; SP, sulfadoxine-pyrimethamine.

We enrolled 196 pregnant women: 103 who received SP for IPTp and 93 who received DP. No statistical differences in demographic characteristics or season of birth were observed between the treatment arms [10]. Serum samples were probed on the PfEMP1 customized array, and cord blood serologic responses to PfEMP1 were compared by treatment arm. There was serorecognition of most protein features on the microarray across the 2 treatment arms (188/259 features; Figure 1).

**Figure 2.**
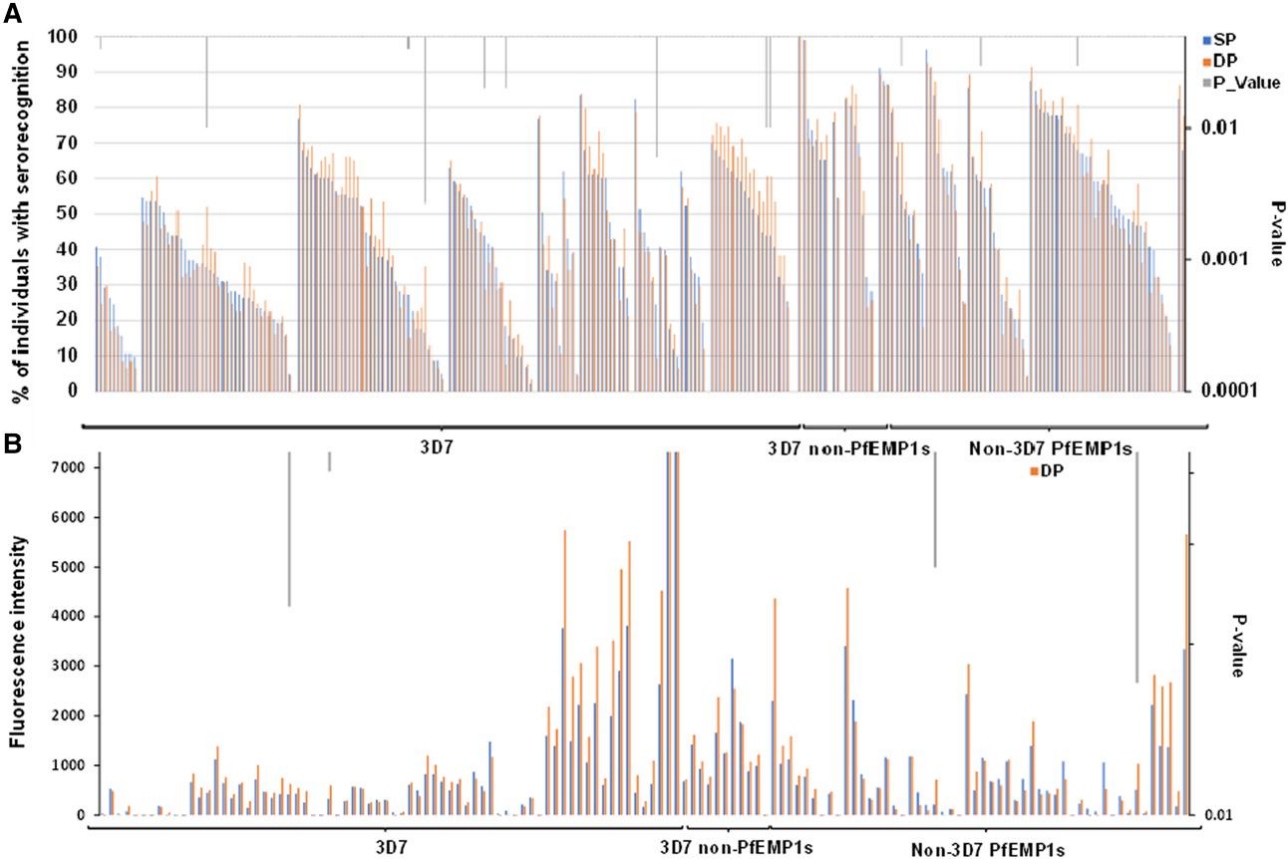
(*A*) Serorecognition of microarray protein features in Malawian children. Protein features included PfEMP1s from the 3D7 reference strain, non-3D7 PfEMP1s, and several non-PfEMP1 malaria antigens. The percentage of individuals with serorecognition is on the y-axis. Columns represent the percentage of individuals with serorecognition for a given protein feature. SP columns are in blue whereas DP columns are in orange. *P* values are displayed on the secondary y-axis in gray. (B) Mean fluorescence intensities to microarray malaria protein features in children born to pregnant women under SP or DP intermittent preventive treatment in pregnancy. Protein features included PfEMP1s from the 3D7 reference strain, non-3D7 PfEMP1s, and several non-PfEMP1 malaria antigens. Fluorescence intensities values for each protein feature are on the y-axis. SP columns are in blue whereas DP columns are in orange. *P* values are displayed on the secondary y-axis in gray. DP, dihydroartemisinin-piperaquine; PfEMP1s, *Plasmodium falciparum* erythrocyte membrane proteins 1; SP, sulfadoxine-pyrimethamine.

Cord blood sera collected from women randomized to SP IPTp recognized 71.0% (184/259) of protein features, while the sera collected from the DP arm recognized 68.3% (177/259) of protein features; the number of serorecognized protein features was not statistically different between the arms (*P* = .38). Serorecognition of more than two-thirds of the same protein features was present across both treatment arms at birth (177/259 features, 68.3%). The SP arm had unique serorecognition of 13 protein features, whereas the DP arm had unique serorecognition of 6 features. The SP- and DP-specific arms included protein features from PfEMP1s known to bind CD36, as well as others that bind endothelial protein C receptor. In addition, the SP-specific arm included 1 feature from VAR2CSA, a PfEMP1 known to bind the placenta that we previously associated with a history of pregnancy [13]. Among the 259 protein features, a higher percentage of individuals in the SP arm serorecognized 8 protein features as compared with the DP arm, and a higher percentage of individuals from the DP arm serorecognized 6 protein features as compared with the SP arm (Figure 2*A*). Overall, despite the serorecognition of a few specific proteins or proteins features, the SP and DP arms had a similar pattern of serorecognition on the protein microarray (κ = 1), suggesting a lack of differential impact of drug selection on serologic responses to PfEMP1s (Figure 2*B*).

## DISCUSSION

In infants born to mothers in a randomized controlled trial of IPTp with SP vs IPTp with DP, we identified no difference in the cord blood serum recognition of diverse PfEMP1 features. This suggests that effectively preventing malaria during pregnancy does not affect the infant's susceptibility to malaria infection early in life when protection is largely mediated by maternal antibodies.

Previous studies have shown that antibodies to PfEMP1s play an important role in the humoral responses against clinical malaria [12], and infants in malaria-endemic regions have high serorecognition of different malaria proteins, including PfEMP1s [14]. In fact, malaria risk early in life is dependent on the immune responses of the mother, and broad PfEMP1 serologic responses acquired early in life may play a major role in conferring protection from uncomplicated malaria [15]. Thus, the signature of seroreactivity against PfEMP1s in infants born to pregnant women randomized to different treatment arms may have important implications for the risk of malaria infection and disease in infants.

A potential limitation is that the PfEMP1s on the microarray may not have exhaustively covered PfEMP1 diversity in Malawi. However, the serorecognition of all the features by the sera from individuals exposed to malaria from Malawi suggests adequate coverage of PfEMP1 diversity. In addition, a single domain of PfEMP1 variants has been used to characterize the immune response to malaria [16].

Our results suggest that 4 to 6 months of highly effective chemoprophylaxis, as conducted during this study, may not significantly change the serologic responses to blood-stage malaria proteins in adult women. This is consistent with previous data suggesting that PfEMP1 serologic responses are long-lasting [15, 17]. This finding also supports our clinical observation that infants born to mothers who received SP vs DP had a similar risk of malaria early in life. This finding alleviates the concern that effective malaria prevention during pregnancy may affect newborns’ capacity to combat malaria infection early in life. Ongoing studies on the dynamics of seroreactivity and recognition of these PfEMP1s in relation to malaria exposure during infants' first year of life may provide critical insights into the acquisition of humoral immunity against PfEMP1s.

## Notes

***Acknowledgments.*** We are grateful to the study participants who allowed us to collect these samples and the study team.

***Author contributions.*** A. O. designed the microarray study, analyzed the data, drafted the manuscript, and revised all drafts of the manuscript. L. R. A. and A. S. conducted and supervised the field study and reviewed the manuscript. M. A. analyzed the data and reviewed the manuscript. R. N., A. Jain, O. T., A. Jasinskas, and P. L. F. designed the microarray, supervised data analysis, and reviewed the manuscript. D. P. M. and J. C. designed and conducted the main study and reviewed the manuscript. M. K. L. designed the main study and the microarray study, helped supervise data analysis, and reviewed the manuscript. M. A. T. designed the microarray study, supervised data analysis, and edited the manuscript. All authors attest that they meet the ICMJE criteria for authorship.

***Patient consent statement.*** Written informed consent was obtained from all study participants prior to the conduct of any study procedure. The study was reviewed and approved by the College of Medicine Research and Ethics Committee, Malawi, and the University of Maryland Institutional Review Board.

***Financial support.*** This work was supported by the United States National Institutes of Health (NIH) (R21AI121990 to M. K. L.); and by the NIH Fundings (K01HL140285; U54CA272205 to A. O., K24AI11499 to M. K. L., and R01HL130750 and R01HL146377 to M. A. T.).
